# Vascular fingerprint tool to identify patients with testicular cancer treated with cisplatin-based chemotherapy at high risk of early cardiovascular events☆

**DOI:** 10.1016/j.esmoop.2024.103631

**Published:** 2024-07-13

**Authors:** A.T. Meuleman, E.L.D. Volders, S. Lubberts, J.M. Kerst, A.N.M. Wymenga, M.J.B. Aarts, M.B. Goncalves, J.D. Lefrandt, G. Steursma, J. Meijer, J. Nuver, J.A. Gietema

**Affiliations:** 1 Department of Medical Oncology, University Medical Center Groningen, University of Groningen, Groningen; 2 Department of Medical Oncology, Netherlands Cancer Institute-Antoni van Leeuwenhoek, Amsterdam; 3 Department of Medical Oncology, Medical Spectrum Twente, Enschede; 4 Department of Medical Oncology, Maastricht University Medical Center, GROW School for Oncology and Developmental Biology, Maastricht, the Netherlands; 5 Department of Medical Oncology, Instituto Português de Oncologia de Lisboa Francisco Gentil (IPOLFG), Lisboa, Portugal; 6 Department of Vascular Medicine, University Medical Center Groningen, University of Groningen, Groningen, The Netherlands

**Keywords:** vascular fingerprint, cardiovascular event, patients with testicular cancer

## Abstract

**Background:**

Patients with testicular cancer treated with chemotherapy have an increased risk of developing early cardiovascular events. Identification of patients with testicular cancer at a high risk of these events enables the development of preventative strategies. This study validates the vascular fingerprint tool to identify these patients.

**Patients and methods:**

We carried out a multicenter prospective study in patients with metastatic testicular cancer [International Germ Cell Cancer Collaborative Group (IGCCCG) good or intermediate risk; retroperitoneal mass <5 cm]. In eligible patients, the vascular fingerprint was assessed before the start of cisplatin-based chemotherapy, which consists of five risk factors, namely, smoking, overweight (body mass index >25 kg/m^2^), hypertension (blood pressure >140/90 mmHg), dyslipidemia (fasting cholesterol >5.1 mmol/l or low-density lipoprotein-cholesterol >2.5 mmol/l), and diabetes mellitus (fasting glucose ≥7.0 mmol/l). The presence of three or more risk factors was defined as high-risk vascular fingerprints. A log-rank test was carried out with a cardiovascular event within 1 year after the start of chemotherapy as the primary endpoint.

**Results:**

A total of 196 patients with metastatic testicular cancer were included; 15 patients (8%) developed a cardiovascular event: 4 (2%) arterial events and 11 (6%) venous thrombotic events. Overall, 189 vascular fingerprint scores were available. Patients with a high-risk vascular fingerprint (62/189) had a higher risk of developing a cardiovascular event (hazard ratio 3.27, 95% confidence interval 1.16-9.18; log-rank: *P* = 0.017). Histological diagnosis, prognosis group, cumulative chemotherapy dose, and retroperitoneal mass size did not differ between patients with or without a cardiovascular event. All patients with an arterial event had a high-risk vascular fingerprint compared with 5/11 patients with a venous event. Overweight was more prevalent in patients with cardiovascular events (87% versus 59%; *P* = 0.037).

**Conclusions:**

The vascular fingerprint is a validated tool to identify patients with testicular cancer at a high risk of developing early cardiovascular events. This tool can be used to develop preventative strategies with anticoagulant treatment.

## Highlights

• 196 patients with metastatic testicular cancer treated with cisplatin-based chemotherapy were prospectively followed.
• A total of 15 patients (8%) developed a cardiovascular event: 4 (2%) arterial events and 11 (6%) venous thrombotic events.
• Patients with a high-risk vascular fingerprint developed a cardiovascular event after chemotherapy more often (HR 3.27).
• The vascular fingerprint is validated to identify patients at high risk and can be used to develop preventative strategies.

## Introduction

In recent decades, the number of testicular cancer (TC) survivors has increased. This is the result of a rise in incidence and excellent survival rates; even in advanced stages, survival rates are >90%, as a result of the introduction of platinum-based chemotherapy in the late 1970s.^1^ Despite excellent cure rates, TC survivors are at an increased risk of developing life-threatening treatment complications during and after treatment, including cardiovascular disease (CVD).^2^ In particular, within the first year after the start of chemotherapy cardiovascular events are serious treatment-related causes of morbidity and mortality.^2^ These can consist of both arterial and venous thromboembolic events. Cardiovascular events during treatment can postpone chemotherapy courses or result in premature termination of chemotherapy, which can lead to less effective cancer treatment. Standard prophylactic anticoagulation therapy is not implemented in current guidelines. The outcomes of previous studies on the effectiveness and safety of prophylactic anticoagulation in patients with TC patients remain uncertain.^3^, ^4^, ^5^ There is an unmet need to identify high-risk patients with TC before the start of chemotherapy in whom prophylactic anticoagulant treatment may prevent cardiovascular events. If patients with TC who are at risk of cardiovascular events can be identified accurately, preventive strategies, such as prophylactic anticoagulation, can be developed and implemented more safely and effectively.

In a small prospective study, Lubberts et al.^6^ developed the ‘vascular fingerprint tool’ that identified patients with TC at high risk of developing cardiovascular events within 1 year after the start of cisplatin-based chemotherapy. The vascular fingerprint is a simple selection tool consisting of five traditional cardiovascular risk factors. The presence of three or more of these risk factors in patients with TC at the start of chemotherapy is defined as a high-risk vascular fingerprint: such patients are considered to be at high risk of developing a cardiovascular event. Lubberts et al.^6^ found that patients with a high-risk vascular fingerprint at the beginning of chemotherapy developed cardiovascular events, especially arterial events, more often within 1 year after the start of chemotherapy than patients with a low-risk vascular fingerprint.

The aim of this study was to validate the vascular fingerprint as a selection tool to identify patients with TC who are at high risk of developing cardiovascular events within the first year after starting cisplatin-based chemotherapy.

## Methods

### Patients

Between June 2015 and May 2021, we carried out a multicenter, prospective, cohort study in patients with disseminated TC in four Dutch hospitals and one hospital in Portugal. All centers were experienced in treating patients with TC. Eligibility criteria were as follows: diagnosed with metastatic TC, classified according to the International Germ Cell Cancer Collaborative Group (IGCCCG) as good- or intermediate prognosis,^7^ <50 years of age at the start of cisplatin-based chemotherapy, and signed informed consent. Exclusion criteria were a history of CVD, a retroperitoneal tumor mass >5 cm, and the use of or indication for anticoagulant therapy at the start of chemotherapy. Patient characteristics before the start of chemotherapy were collected to assess the vascular fingerprint score. Four to six weeks after the last course of chemotherapy, treatment response was assessed by evaluating tumor marker normalization and a computed tomography scan of the chest and abdomen. Based on the restaging, it was decided whether additional treatment, such as retroperitoneal lymph node dissection, was necessary. One year after the start of chemotherapy, any cardiovascular events or relapses were registered, including arterial and venous vascular events [World Health Organization (WHO) ICD-10 I1–99].

### Vascular fingerprint score

**Figure 1:**
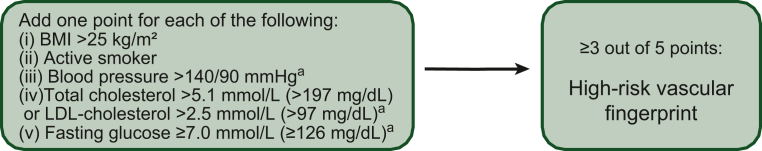
**High-risk vascular fingerprint.** BMI, body mass index; LDL, low-density lipoprotein. ^a^Or use of anti-hypertensive, lipid-lowering or blood glucose-lowering drugs.

During regular clinical care before the start of chemotherapy, the patient’s medical history, disease and treatment characteristics, smoking behavior, and medication use were assessed. Weight, height, and blood pressure (using a sphygmomanometer) were measured during a physical examination. A single fasting blood sample was drawn to determine total cholesterol, low-density lipoprotein (LDL)-cholesterol, and glucose. The vascular fingerprint score consists of five traditional cardiovascular risk factors, namely, overweight, smoking, hypertension, dyslipidemia, and impaired glucose tolerance (Figure 1).^6^ The maximum vascular fingerprint score is 5; 1 point for each of the aforementioned risk factors. Overweight was defined as a body mass index (BMI) >25 kg/m^2^; smoking as active smoking at the start of chemotherapy or smoking an average of one or more cigarettes per day during the past year; hypertension as a blood pressure of >140/90 mmHg or use of antihypertensive drugs; dyslipidemia as a total cholesterol of >5.1 mmol/l or LDL-cholesterol of >2.5 mmol/l or use of lipid-lowering drugs; and impaired glucose tolerance as fasting glucose of ≥7 mmol/l or use of blood glucose-lowering drugs. A score of ≥3 was defined as a high-risk vascular fingerprint.^6^

### Khorana score, coagulation factor VIII, and lactate dehydrogenase

The Khorana score, a known and validated score that predicts the chance of developing venous thromboembolism in patients with all types of cancer, was also calculated.^8^ Lactate dehydrogenase (LDH), a known risk factor for cardiovascular events, and coagulation factor VIII (FVIII), an essential blood-clotting protein, were also measured.

### Cardiovascular events

The following cardiovascular events that occurred within 1 year after the start of chemotherapy were registered: myocardial infarction (WHO ICD-10, I20-I25), ischemic cerebrovascular accidents (WHO ICD-10, I63-I66 and G45), infarction in other specific organ systems (WHO ICD-10, K76.3, K55, D73.5, M62.2, and N28.0), and venous thromboembolic events (WHO ICD-10, I26, and I81-82). Thrombophlebitis (WHO ICD-10 I80) was also registered but not defined as an event aimed to identify with the vascular fingerprint because it is superficial with often clinically mild presentation and without a clear indication for therapeutic anticoagulant therapy. However, in our study, some patients received anticoagulant therapy for thrombophlebitis. This was registered because of a presumed lower risk of developing a more severe cardiovascular event after treatment with anticoagulant therapy.

### Statistical analysis

Based on the article of Lubberts et al.,^6^ 19% of patients with TC with a high-risk vascular fingerprint developed an arterial cardiovascular event, compared with 2% of patients without a high-risk vascular fingerprint. To confirm the difference (17%) between these groups, and thus validate the vascular fingerprint as a selection tool, 155 patients should be included to reach 80% power (α = 0.05, two-sided testing), assuming a 1 : 4 distribution between the groups. Estimating that 15% of patients would be nonassessable, a total minimum number of 178 patients was needed.

For the demographic, clinical, and laboratory characteristics of the patients that were normally distributed, means and standard deviations were used. If characteristics were not normally distributed, medians and ranges were used. To compare differences between groups, Mann–Whitney *U* tests were used when comparing groups with continuous data that were not normally distributed, and unpaired *t*-tests for normally distributed data. The chi-square test (or Fisher’s exact test when expected counts were <5) was used when comparing categorical variables between groups.

A log-rank analysis was carried out to determine whether patients with TC with a high-risk vascular fingerprint had a higher risk of developing a cardiovascular event. This analysis was carried out first for all cardiovascular events, and then separately for arterial and venous events. Patients who developed thrombophlebitis and received anticoagulant treatment were allocated to the noncardiovascular event group and censored at the beginning of anticoagulant treatment.

In an additional analysis, age and FVIII were added as risk factors to the vascular fingerprint tool. This was done with receiver operating characteristic curves and evaluated using sensitivity, specificity, and area under the curve (see Supplementary Methods, available at https://doi.org/10.1016/j.esmoop.2024.103631).

*P* values were reported as two-sided and considered significant when <0.05. All data were analyzed with SPSS statistics 23.0 (IBM Inc., Chicago, IL).

### Ethics Approval and trial registration

Procedures in this study were carried out in compliance with relevant laws and institutional guidelines and were reviewed by the Ethical Approval Committee of the University Medical Center Groningen on 10 September 2015 (reference number: METC 2015/399). Written informed consent of all participants was obtained. The study is registered in ClinicalTrials.gov (NCT02573584).

## Results

### Patients

In total, 196 patients with metastasized TC were included and available for data analysis. Patient demographic, clinical, and laboratory characteristics are shown in Table 1. Sixty-two patients (62/189, 33%) had a high-risk vascular fingerprint and 127 patients had a low-risk vascular fingerprint (127/189, 67%; Supplementary Table S1, available at https://doi.org/10.1016/j.esmoop.2024.103631). From seven patients the vascular fingerprint could not be calculated because of partially missing data. As shown in Table 1, patients who developed a cardiovascular event were older and had higher levels of total cholesterol, LDL-cholesterol, and glucose compared with patients who did not develop a cardiovascular event. No significant differences were found regarding histological diagnosis; IGCCCG prognosis group; size of retroperitoneal mass; BMI; blood pressure; FVIII; LDH; and cumulative dose of cisplatin, bleomycin, and etoposide. The Khorana score did not differ between both groups and was not predictive for the development of both arterial and venous events combined, and for venous events only (data not shown). None of the patients died within 1 year after starting chemotherapy.

**Table 1:** Patient demographic, clinical, and laboratory characteristics (*n* = 196)

Characteristics	Total (*n* = 196)	Cardiovascular event (*n* = 15)	No cardiovascular event (*n* = 181)^a^	*P*^b^
Age at the start of chemotherapy, years, median (range)	32 (17-50)	40 (22-50)	31 (17-48)	0.013
Histological diagnosis, *n* (%)				0.765
Nonseminoma	141 (72)	10 (67)	131 (72)	
Seminoma	55 (28)	5 (33)	50 (28)	
IGCCCG prognosis group, *n* (%)				0.611
Good	181 (92)	15 (100)	166 (92)	
Intermediate	15 (8)	0 (0)	15 (8)	
Chemotherapy (cumulative dose), median (range)				
Cisplatin mg	695 (195-1100)	815 (460-980)	685 (195-1100)	0.202
Bleomycin USP	270 (0-360)	180 (0-270)	270 (0-360)	0.087
Etoposide mg	3388 (2300-5550)	4090 (2300-4915)	3360 (2460-5550)	0.158
Ifosfamide mg	0 (0-53460)	0 (0)	0 (0-53460)	0.475
Carboplatin mg	0 (0-1912)	0 (0)	0 (0-1912)	0.683
Maximum diameter of retroperitoneal mass, median (range)	1.7 (0.0-4.8)	1.9 (1.0-2.6)	1.7 (0.0-4.8)	0.696
<3.5 cm, *n* (%)	186 (95)	15 (100)	171 (94)	>0.99
>3.5 cm and <5.0 cm, *n* (%)	10 (5)	0 (0)	10 (6)	>0.99
RPLND, *n* (%)				
Before chemotherapy	1 (1)	0 (0)	1 (1)	>0.99
After chemotherapy	24 (12)	2 (13)	22 (12)	>0.99
Oncological status after chemotherapy, *n* (%)				0.201
Remission	185 (94)	13 (87)	171 (95)	
Relapse after remission	11 (6)	2 (13)	9 (5)	
Blood pressure (mmHg)				
Systolic				
Mean	132	135	131	0.354
SD	13	15	13	
Diastolic				
Mean	78	81	78	0.277
SD	11	8	11	
BMI (kg/m^2^), median (range)	26 (17-54)	28 (21-36)	26 (17-54)	0.051
Total cholesterol (mmol/l)				0.018
Mean	4.7	5.2	4.6	
SD	1.0	1.1	0.9	
LDL cholesterol (mmol/l), median (range)	3.0 (1.4-5.3)	3.7 (2.2-5.1)	3.0 (1.4-5.3)	0.005
Serum glucose (mmol/l), median (range)	5.4 (4.0-8.6)	5.7 (5.1-7.2)	5.0 (4.0-8.6)	0.014
FVIII, %^c^				0.408
Mean	150	160	148	
SD	40	29	41	
LDH, U/l				0.592
Mean	227	208	228	
SD	132	57	136	

BMI, body mass index; FVIII, factor VIII; IGCCCG, International Germ Cell Cancer Collaborative Group; LDH, lactate dehydrogenase; LDL, low-density lipoprotein; RPLND, retroperitoneal lymph node dissection; SD, standard deviation; USP, United States Pharmacopeia.

a One patient in this study had a central vascular access device and did not develop a cardiovascular event.

b Mann–Whitney *U* test/chi-square test/Fisher’s exact test/Student’s *t*-test.

c FVIII was only available in patients from the University Medical Center Groningen (*n* = 85).

### Cardiovascular events

As shown in Table 2, 15 of 196 (8%) patients developed a cardiovascular event, of which 4 (2%) developed an arterial event and 11 (6%) developed a deep venous thrombotic event (thrombophlebitis not included). All events occurred during chemotherapy treatment, except for one that occurred 3 weeks after completion of chemotherapy. A total of 6 out of 196 patients (3%) developed thrombophlebitis (WHO ICD-10, I80) for which they received anticoagulant treatment in the form of therapeutic low-molecular-weight heparin for up to 6 weeks. One patient who developed a cardiovascular event (arterial event during the second cycle of etoposide- and cisplatin-containing chemotherapy) had a relapse of TC, 8 months after completion of chemotherapy treatment for which he received second-line chemotherapy.

**Table 2:** Characteristics of patients with vascular events (*n* = 15)

No.	Age (years)	Event	Symptoms	High-risk vascular fingerprint	Risk factors	Axial mean largest retroperitoneal mass on CT scan	Timing event in days after the start of chemotherapy	Timing	Treatment	Outcome
**Arterial events**										
1	47	Ischemic stroke	Yes	Yes	Overweight, hypertension, and dyslipidemia	1.5 cm	51	During the third-cycle BEP	Anticoagulation	Mild residual symptoms
2	40	Thrombosis superior mesenteric artery	Yes	Yes	Smoker, overweight, and dyslipidemia	2.4 cm	27	During the first-cycle EP	Surgery and anticoagulation	Terminal stoma
3	45	Ischemic stroke	Yes	Yes	Overweight, hypertension, and dyslipidemia	1.7 cm	28	During the second-cycle BEP	Medication only	Died 14 months after the start of chemotherapy
4	50	Acute vascular occlusion common iliac artery left	Yes	Yes	Smoker, overweight, and dyslipidemia	2.1 cm	29	During the second-cycle EP	Surgery and anticoagulation	Stent in the left common iliac artery left and underwent TIP chemotherapy because of relapse 8 months after completion of EP
**Venous events**										
1	39	DVT upper extremity	Yes	No	Overweight and dyslipidemia	1.7 cm	42	During the third-cycle BEP	Anticoagulation	Full recovery
2	22	Pulmonary embolism	Yes	No	Overweight and dyslipidemia	2.1 cm	31	During the second-cycle BEP	Anticoagulation	Full recovery
3	42	Pulmonary embolism and thrombophlebitis	Yes	No	Smoking and dyslipidemia	1.9 cm	14	During the first-cycle BEP/EP	Anticoagulation	Full recovery
4	43	Pulmonary embolism	Yes	No	None	1.9 cm	43	During the third-cycle BEP/EP	Anticoagulation	Full recovery
5	49	Pulmonary embolism	Yes	Yes	Overweight, hypertension, and dyslipidemia	1.7 cm	49	During the third-cycle EP	Anticoagulation	Full recovery


**No.**	**Age, years**	**Event**	**Symptoms**	**High-risk vascular fingerprint**	**Risk factors**	**Axial mean largest retroperitoneal mass on CT scan**	**Timing in days after the start of chemotherapy**	**Timing**	**Treatment**	**Outcome**
6	25	Pulmonary embolism	Yes	Yes	Smoker, overweight, hypertension, and dyslipidemia	2.4 cm	53	During the third-cycle EP	Anticoagulation	Full recovery
7	24	Pulmonary embolism	Yes	No	Overweight and dyslipidemia	1.5 cm	38	During the second-cycle BEP/EP	Anticoagulation	Full recovery
8	24	DVT lower extremity	Yes	Yes	Overweight, hypertension, and dyslipidemia	1.0 cm	50	During the third-cycle BEP	Anticoagulation	Full recovery
9	45	Pulmonary embolism and thrombosis inferior vena cava	Yes	No	Overweight and dyslipidemia	2.6 cm	93	25 days after completion of EP chemotherapy	Anticoagulation	Full recovery
10	38	DVT arm	Yes	Yes	Smoker, overweight, hypertension, and high glucose	1.1 cm	62	During the fourth-cycle EP	Anticoagulation	Full recovery
11	38	DVT arm	Yes	Yes	Overweight, hypertension, and dyslipidemia	1.9 cm	33	During the second-cycle BEP	Anticoagulation	Full recovery

BEP, bleomycin-, etoposide-, and cisplatin-containing chemotherapy; CT, computed tomography; DVT, deep venous thrombosis; EP, etoposide- and cisplatin-containing chemotherapy; TIP; paclitaxel-, ifosfamide-, and cisplatin-containing chemotherapy.

### The vascular fingerprint, cardiovascular events, and cardiovascular risk factors

**Figure 2:**
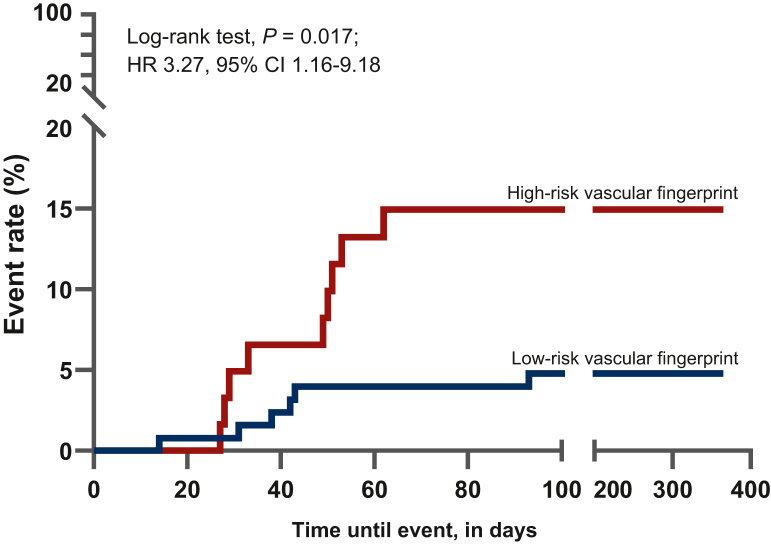
Time-to-event analysis including arterial and venous events.

Patients with TC with a high-risk vascular fingerprint had a higher risk of developing a cardiovascular event [log-rank test: *P* = 0.017; hazard ratio (HR) 3.27; 95% confidence interval (CI) 1.16-9.18; Figure 2]. Patients with a high-risk vascular fingerprint developed a cardiovascular event more often than patients with a low-risk vascular fingerprint [9/62 (15%) versus 6/127 (5%); chi-square test: *P* = 0.041]. Of the five cardiovascular risk factors, only overweight (BMI >25 kg/m^2^) was significantly more present in the group with a cardiovascular event (87% versus 59%; *P* = 0.037, Table 3).

**Table 3:** Differences in cardiovascular risk factors in patients with versus without vascular events

Differences in cardiovascular risk factors	Cardiovascular events (*n* = 15)	No cardiovascular events (*n* = 181), median (range)	*P*^a^
Age at start of chemotherapy, years, median (range)	40 (22-50)	31	0.013
Age at vascular event, years, median (range)	40 (22-50)	NA	NA
Time to event, days, median (range)	42 (14-93)	NA	NA
Cardiovascular risk factors before the start of chemotherapy, *n*/*N* (%)			
Current smoker	5/15 (33)	63/181 (35)	0.908
Overweight (BMI >25 kg/m^2^)	13/15 (87)	107/180 (59)	0.037
Hypertension^b^	7/15 (47)	47/177 (27)	0.132
Dyslipidemia^c^	13/15 (87)	113/161 (70)	0.238
Diabetes mellitus^d^	1/15 (7)	6/174 (3)	0.445
FVIII >150%, *n*/*N* (%)	6/10 (60)	35/75 (47)	0.511
Total score vascular fingerprint, median (range)	3 (0-4)	2 (0-4)	0.035
High-risk vascular fingerprint^e^, *n*/*N* (%)	9/15 (60)	53/174 (31)	0.041
Total score Khorana score, median (range)	1 (1-2)	1 (1-4)	0.519
High-risk Khorana^f^, *n*/*N* (%)	0/14 (0)	3/173 (2)	>0.99
High-risk Khorana^g^, *n*/*N* (%)	3/14 (21)	20/173 (12)	0.387

BMI, body mass index; FVIII, coagulation factor VIII; NA, not applicable.

a Mann–Whitney *U* test/chi-square test/Fisher’s exact test/linear-by-linear association test.

b Hypertension is defined as systolic blood pressure >140 mmHg and/or diastolic blood pressure >90 mmHg or use of antihypertensive medication.

c Dyslipidemia is defined as fasting total cholesterol >5.1 mmol/l, low-density lipoprotein >2.5 mmol/l, or the use of lipid-lowering medication.

d Diabetes mellitus is defined as fasting glucose ≥7.0 mmol/l or the use of blood glucose-lowering medication.

e High-risk vascular fingerprint is defined as ≥3 out of 5 points (also see the ‘Methods’ section). From seven patients, the vascular fingerprint could not be calculated because of partially missing data.

f High-risk Khorana score is defined as ≥3 out of 5 points.

g High-risk Khorana score is defined as ≥2 out of 5 points.

All patients with an arterial event had a high-risk vascular fingerprint [4/62 (6%) versus 0/127 (0%); *P* = 0.011]; log-rank analysis for arterial events only was also significant (log-rank test: *P* = 0.004). Patients with a venous event did not have a high-risk vascular fingerprint significantly more often [5/62 (8%) versus 6/127 (5%); *P* = 0.345] and the log-rank test for venous events only was not significant (log-rank test: *P* = 0.310). Patients with an arterial event were older than those with a venous event [median 46 years (range 40-50 years) versus median 38 years (range 22-49 years); *P* = 0.040]. Differences in cardiovascular risk factors between patients with an arterial or venous event are summarized in Supplementary Table S2, available at https://doi.org/10.1016/j.esmoop.2024.103631.

### Adding age or FVIII to the vascular fingerprint score

The cut-off value for age with the Youden Index method was 38 years. Adding age >38 years to the vascular fingerprint and defining three or more out of six risk factors as a high-risk vascular fingerprint resulted in the detection of more cardiovascular events (see Supplementary Table S3, available at https://doi.org/10.1016/j.esmoop.2024.103631). When the vascular fingerprint included age, the log-rank analysis was more significant than the original fingerprint (log-rank: *P* = 0.002; HR 6.03, 95% CI 1.70-21.37 versus log-rank: *P* = 0.017; HR 3.27; 95% CI 1.16-9.18). Adding FVIII levels >150% before chemotherapy (*n* = 85, available in only one center) as a risk factor to the vascular fingerprint and defining the presence of three or more out of six risk factors as a high-risk vascular fingerprint resulted in the detection of more cardiovascular events in this center (Supplementary Table S3, available at https://doi.org/10.1016/j.esmoop.2024.103631).

## Discussion

Patients with disseminated TC treated with cisplatin-based chemotherapy have an increased risk of developing cardiovascular events. In this validation study, the vascular fingerprint tool was shown to be effective in upfront identifying patients with TC at high risk for developing a cardiovascular event within the first year after the start of chemotherapy. Patients with a high-risk vascular fingerprint had a three times higher risk of developing a cardiovascular event. The vascular fingerprint tool is particularly effective in identifying patients at high risk of arterial events.

The proportion of patients with cardiovascular events in our study was relatively small. A previous retrospective single-center study describing six different studies reported that the percentage of patients with TC with (mainly venous) thromboembolic events was between 5.2% and 26.5%.^9^ The relatively small number of events in our study could be explained by the exclusion of patients with TC with a large retroperitoneal mass (>5 cm) and a poor prognosis, both known risk factors for cardiovascular events.^4^ By not including these patients in the current study, the vascular fingerprint tool could especially be validated in patients that were previously not qualified as high risk. Furthermore, the original study by Lubberts et al.^6^ was carried out in one single center in which the study population could have had a worse cardiovascular risk profile before treatment. Despite the relatively low number of events in our study, the vascular fingerprint appeared to be a useful tool to identify patients at high risk for cardiovascular events.

The current study showed that being overweight is an important risk factor for developing a cardiovascular event. Being overweight is a fundamental cause of other cardiovascular risk factors and often results in hypertension, dyslipidemia, and diabetes at a later stage.^10^ As a substantial number of patients with TC develop overweight and metabolic syndrome within 5 years after the start of chemotherapy, it is important to implement lifestyle interventions (eating a healthy diet, being more physically active, and quitting smoking) as early as possible.^11^ Unfortunately, because of the lack of time between diagnosis and the start of chemotherapy, its short-term benefits on the development of cardiovascular events during and shortly after chemotherapy would be limited.

The results indicated that the vascular fingerprint is especially useful in identifying patients at high risk for developing arterial events. The pathophysiology of arterial events differs from that of venous events. Arterial events are caused by vascular endothelial damage and high-shear stress, whereas venous events are a result of stasis and hypercoagulability.^12^ Currently, routine use of prophylactic anticoagulation for patients with disseminated TC during chemotherapy is not recommended because it is unclear which patients are at high risk of developing thromboembolic events.^13^^,^^14^ The updated European Society for Medical Oncology (ESMO) clinical treatment guideline of 2022 for TC recommends practitioners to consider prophylactic anticoagulation for patients with metastatic TC who have a retroperitoneal lymph node >3.5 cm, stage III disease, or are classified as poor risk.^13^ The European Association of Urology (EAU) guidelines only provide a general recommendation; for individual patients with TC, the potential benefits and risks of prophylactic anticoagulation to prevent thromboembolic events should be considered.^14^ The studies cited in the EAU guidelines mainly concerned thromboprophylaxis for venous thromboembolic events in various cancer types.^15^, ^16^, ^17^, ^18^ Both the ESMO and EAU guidelines recommend avoiding vascular access devices whenever possible.^13^^,^^14^ In our study, no poor-risk patients were included, only 10 patients had a retroperitoneal mass between 3.5 and 5 cm, and 1 patient had a central vascular access device, but none of these developed a cardiovascular event. Other known risk factors for developing cardiovascular events in patients with TC are elevated LDH levels and a high Khorana score,^19^, ^20^, ^21^ but both risk factors were not discriminatory for the development of cardiovascular events in this study.

Previous studies on the effectiveness and safety of prophylactic anticoagulation in patients with TC remain scarce, and the results have been contradictory.^3^, ^4^, ^5^^,^^19^^,^^22^ Arguments in favor of administering prophylactic anticoagulation to high-risk patients with TC were that a significant decrease in thromboembolic events was achieved and that its benefit outweighed the risk of bleeding.^4^^,^^5^^,^^19^ Arguments against thromboprophylaxis were that it did not reduce the frequency of thrombosis,^3^^,^^22^ but instead resulted in more bleeding complications.^3^ A review by Meng et al.^4^ described a large variety of risk factors for thromboembolic events during platinum-based chemotherapy, which shows that it is challenging to identify patients with TC at a high risk of a cardiovascular event. Our vascular fingerprint can identify patients with TC at a higher risk, especially arterial events, so it could be used to adequately select patients for treatment strategies to prevent cardiovascular events.

When selecting patients with TC for prophylactic anticoagulation therapy in future clinical trials, it should be considered that the pathophysiology of arterial events differs from that of venous events.^12^ Both of these events can have a major impact on the patients, their treatment, and their quality of life afterward. The treatment of arterial events is different from that of venous events. Arterial events are usually treated with various types of anticoagulants (e.g. direct oral anticoagulants, antiplatelet drugs, or vitamin K antagonists), whereas venous events are often treated with low-molecular-weight heparins.^23^ Prophylactic treatment with statins could also be considered in patients with TC because it is known to effectively prevent cardiovascular events and it can be used in conjunction with anticoagulant treatment.^24^^,^^25^ In the recently updated American Society of Clinical Oncology (ASCO) clinical guideline of 2023 for patients with cancer, the direct factor Xa inhibitors, apixaban and rivaroxaban, were added as options for extended thromboprophylaxis after cancer surgery.^26^ This guideline also suggests consideration of apixaban, rivaroxaban, or low-molecular-weight heparin as optional prophylaxis for high-risk outpatients with cancer, after individual discussions about relative benefits and harms.^26^ In future trials, it is of high importance to identify an optimal combination of prophylactic anticoagulation therapy to reduce the risk of both arterial and venous events.

To our knowledge, no studies have been published on physical long-term outcomes in patients with TC who developed a cardiovascular event during chemotherapy treatment. It is known that patients with TC who have developed CVD reported impaired quality of life in physical domains.^27^ These results emphasize the importance of preventing cardiovascular events in patients with TC.

Our study and the vascular fingerprint tool have several strengths. First, the vascular fingerprint tool can be easily implemented in routine clinical care because no additional protocols or expensive measurements are needed. Second, we conducted a multicenter study, which improves the generalizability of the vascular fingerprint tool. A potential limitation of this study is the presence of thrombophlebitis in six patients who were treated with anticoagulants. As these patients received anticoagulants, it was unlikely that they would develop a major cardiovascular event thereafter. However, a log-rank analysis accounted for this potential confounding. Another confounder is age, a known risk factor for cardiovascular events that was not included in the vascular fingerprint tool. It might be needed to approach patients with TC at an older age differently regarding preventive strategies because they are more likely to develop arterial events.

In conclusion, the vascular fingerprint is a simple useful tool and has now been validated to identify patients with disseminated TC who are at high risk for developing cardiovascular events during or after cisplatin-based chemotherapy. Being overweight is an important component of the vascular fingerprint and thereby an early risk factor for developing a cardiovascular event. Therefore, lifestyle changes should be advised, but their benefits before the start of chemotherapy would be limited. A high-risk vascular fingerprint at the start of chemotherapy adequately selects patients with TC at a high risk of developing cardiovascular events who might benefit from interventions to prevent such events. Future clinical trials should evaluate lifestyle interventions and the use of prophylactic anticoagulants in these identified high-risk patients.
