# Supplementary material for: Vascular fingerprint tool to identify patients with testicular cancer treated with cisplatin-based chemotherapy at high risk of early cardiovascular events
Source: ESMO Open. 2024 Jul 13;9(7):103631. doi: 10.1016/j.esmoop.2024.103631 (PMC11298865; doi:10.1016/j.esmoop.2024.103631)
Supplement: Supplementary data [file mmc1.docx]

**Supplementary Materials**

1. **Supplementary methods**

The five participating hospitals were: 1. University Medical Center Groningen (UMCG), Groningen (*n* = 99) 2. Netherlands Cancer Institute - Antoni van Leeuwenhoek (NKI-AVL), Amsterdam (*n* = 60), 3. Maastricht University Medical Centre (MUMC), Maastricht (*n* = 8) 4. Medical Spectrum Twente (MST), Enschede (*n* = 14) (all in the Netherlands), and 5. Instituto Português de Oncologia de Lisboa Francisco Gentil (IPOLFG), Lisbon, Portugal (*n* = 15). All centers were experienced in treating patients with TC.

- 1. *Age and FVIII included in vascular fingerprint score as post-hoc analyses*

First, ROC curves with the original vascular fingerprint score and age at start of chemotherapy as factors were visualized. The Youden Index method was used to determine the cut-off value for age with the highest combined specificity and sensitivity. This cut-off value for age was added to the vascular fingerprint. A point was added to the total vascular fingerprint score when a patient’s age was above this value. Analyses were done with either ≥3 or ≥4 out of 6 risk factors defined as high-risk vascular fingerprint.

Second, the original vascular fingerprint and the modified vascular fingerprint including age were evaluated with ROC curves, and both fingerprint scores were compared by calculating sensitivity and specificity, and performing a log-rank analysis for each modified vascular fingerprint.

In a different analyses, FVIII was added to the original vascular fingerprint score. A point was added when a patient had a FVIII >150%. Analyses were done with ≥3 out of 6 risk factors defined as high-risk vascular fingerprint. A log-rank analysis was performed for the modified vascular fingerprint.

1. **Supplementary results**
   1. *Age and FVIII included in vascular fingerprint score*

Adding age ≥38 years to the vascular fingerprint resulted in the detection of more cardiovascular events, defining ≥3 out of 6 risk factors as high-risk vascular fingerprint.
79 patients (*n* = 79/188 [40%]) then had a high-risk vascular fingerprint, of whom 12 patients (*n* = 12/79 [15%]) developed cardiovascular events, compared to three cardiovascular events in the 109 patients (3%) with a low-risk vascular fingerprint (*p*=0.002) (Table S3).

FVIII was only available in patients treated at the UMCG. Adding FVIII >150% resulted in the detection of 53 patients with a high-risk vascular fingerprint. 7 patients with a cardiovascular event had a high-risk vascular fingerprint (7/10 [70%]) compared to 46 patients without a cardiovascular event (46/87 [53%], *p*=0.340 (Table S3).

| **Table S1. Patients with high-risk vascular fingerprint versus low-risk vascular fingerprint before start of chemotherapy** | | | | | | | |
| --- | --- | --- | --- | --- | --- | --- | --- |
|  | High-risk vascular fingerprint  n = 62 | |  | Low-risk vascular fingerprint  n = 127 | |  | *P*^a^ |
|  | Median/n | Range/% |  | Median/n | Range/% |  |  |
| Age at start of chemotherapy, years | 36 | 18 – 50 |  | 29 | 17 – 47 |  | **<0.001** |
| Cardiovascular events  *Arterial events*  *Venous events* | 9/62  4/62  5/62 | 15  6  8 |  | 6/127  0/127  6/127 | 5  0  5 |  | **0.041**  **0.011**  0.345 |
| Cardiovascular risk factors at start of chemotherapy  *Current smoker*  *Overweight (BMI >25 kg/m^2^)*  *Hypertension^b^*  *Dyslipidemia^c^*  *Diabetes Mellitus^d^* | 38/62  62/62  35/61  58/60  4/57 | 61  100  57  97  7 |  | 26/127  54/127  17/125  66/114  3/126 | 21  43  14  58  2 |  | **<0.001**  **<0.001**  **<0.001**  **<0.001**  0.207 |
| Total score Khorana score | 1.0 | 0 – 3 |  | 1.0 | 0 – 4 |  | **0.040** |
| High-risk Khorana^e^ | 12/59 | 20 |  | 9/122 | 7 |  | **0.011** |
| Abbreviations: BMI, body mass index. ^a^ Mann-Whitney U test / Chi-square test / Fisher exact test / Linear-by-linear Association test.  ^b^ Hypertension defined as systolic blood pressure > 140 mmHg and/or a diastolic blood pressure >90 mmHg or use of antihypertensive medication.  ^c^ Dyslipidemia defined as fasting total cholesterol >5,1 mmol/L, low-density lipoprotein >2,5 mmol/L or use of lipid-lowering medication.  ^d^ Diabetes mellitus is defined as a fasting glucose ≥7.0 mmol/L or use of blood glucose lowering medication.  ^e^ High-risk Khorana score defined as ≥ 2 out of 5 points. | | | | | | | |

| **Table S2. Differences in cardiovascular risk factors in patients with an arterial event versus venous event** | | | | | | | | | |
| --- | --- | --- | --- | --- | --- | --- | --- | --- | --- |
|  | Cardiovascular events  (n = 15) | | Arterial events  (n = 4) | |  | Venous events  (n = 11) | |  | *P*^a^ |
|  | Median/n | Range/% | Median/n | Range/% |  | Median/n | Range/% |  |  |
| Age at start of chemotherapy, years | 40 | 22 – 50 | 46 | 40 – 50 |  | 38 | 22 – 49 |  | **0.040** |
| Age at vascular event, years | 40 | 22 – 50 | 46 | 40 – 50 |  | 38 | 22 – 49 |  | **0.040** |
| Time to event, days | 42 | 14 – 93 | 29 | 27 – 51 |  | 43 | 14 – 93 |  | 0.177 |
| Cardiovascular risk factors before start of chemotherapy  *Current smoker*  *Overweight (BMI>25 kg/m^2^)*  *Hypertension^b^*  *Dyslipidemia^c^*  *Diabetes Mellitus^d^* | 5/15  13/15  7/15  13/15  1/15 | 33  87  47  87  7 | 2/4  4/4  2/4  4/4  0/4 | 50  100  50  100  0 |  | 3/11  9/11  5/11  9/11  1/11 | 27  82  46  82  9 |  | 0.560  1.000  1.000  1.000  1.000 |
| FVIII > 150% | 6/10 | 60 | 2/3 | 67 |  | 4/7 | 57 |  | 1.000 |
| Total score vascular fingerprint | 3 | 0 – 4 | 3 | 3 – 3 |  | 2 | 0 – 4 |  | 0.343 |
| High-risk vascular fingerprint^e^ | 9/15 | 60 | 4/4 | 100 |  | 5/11 | 46 |  | 0.103 |
| Total score Khorana score | 1 | 1 – 2 | 1 | 1 – 2 |  | 1 | 1 – 2 |  | 0.585 |
| High-risk Khorana^f^ | 3/14 | 0 | 1/3 | 33 |  | 2/11 | 18 |  | 1.000 |
| Abbreviations: BMI, body mass index; FVIII, Factor VIII. ^a^ Comparing arterial events with venous events. Used tests: Mann-Whitney U test / Chi-square test / Fisher exact test / Linear-by-linear Association test.  ^b^ Hypertension defined as systolic blood pressure > 140 mmHg and/or a diastolic blood pressure >90 mmHg or use of antihypertensive medication.  ^c^ Dyslipidemia defined as fasting total cholesterol >5,1 mmol/L, low-density lipoprotein >2,5 mmol/L or use of lipid-lowering medication.  ^d^ Diabetes mellitus is defined as a fasting glucose ≥7.0 mmol/L or use of blood glucose lowering medication.  ^e^ High-risk vascular fingerprint defined as ≥ 3 out of 5 points (also see method section). ^f^ High-risk Khorana score defined as ≥ 2 out of 5 points. | | | | | | | | | |

| **Table S3. Comparisons of different vascular fingerprint scores in patients with versus without cardiovascular events** | | | | | | | |
| --- | --- | --- | --- | --- | --- | --- | --- |
|  | Cardiovascular events  (n = 15) | |  | No cardiovascular events  (n = 181) | | *P*^a^ | *P*^b^ |
|  | Median/n | Range/% |  | Median/n | Range/% |  |  |
| Total score vascular fingerprint | 3.0 | 0 – 4 |  | 2.0 | 0 – 4 | **0.036** |  |
| High-risk vascular fingerprint | 9/15 | 60.0 |  | 53/174 | 30.5 | **0.041** | **0.017** |
| Total score vascular fingerprint, including age | 4.0 | 1 – 5 |  | 2.0 | 0 – 5 | **0.002** |  |
| High-risk vascular fingerprint, including age, 3 factors | 12/15 | 80 |  | 66/172 | 39 | **0.002** | **0.001** |
| High-risk vascular fingerprint, including age, 4 factors | 8/15 | 53 |  | 29/172 | 17 | **0.003** | **<0.001** |
| Total score vascular fingerprint (UMCG only) | 3 | 0 – 4 |  | 2 | 0 – 4 | 0.723 |  |
| High-risk vascular fingerprint (UMCG only) | 5/10 | 50 |  | 35/87 | 40 | 0.736 | 0.556 |
| Total score vascular fingerprint, including FVIII (UMCG only) | 3 | 1 – 4 |  | 3 | 0 – 5 | 0.502 |  |
| High-risk vascular fingerprint, including FVIII (UMCG only) | 7/10 | 70 |  | 46/87 | 53 | 0.340 | 0.298 |
| Abbreviations; BMI, body mass index; FVIII, Factor VIII  ^a^ Mann-Whitney U test / Chi-square test / Fisher exact test / Linear-by-linear Association test  ^b^ Log-rank test | | | | | | | |
